# Incidence and Trends in Paediatric Trampoline-Related Injuries

**DOI:** 10.7759/cureus.94250

**Published:** 2025-10-09

**Authors:** Karim Saltajai, Michael Maxwell, Tharaga Kirupakaran, Michael Jiang, Freya Bakko, Benjamin Zakaria, Ritesh Sharma

**Affiliations:** 1 Trauma and Orthopaedics, Epsom and St Helier University Hospitals NHS Trust, London, GBR

**Keywords:** fracture, incidence, injury, paediatric, soft tissue, trampoline

## Abstract

Introduction: Trampolining in the United Kingdom (UK) has seen a major increase in popularity among children, leading to a rising incidence of associated injuries. While international studies have described the injury patterns, recent UK-specific epidemiological data are limited. This study investigates the incidences and trends of trampoline-related orthopaedic injuries within a UK NHS Trust.

Methods: A retrospective observational study was conducted at a Trust with two district general hospitals. Electronic medical records were analysed for all patients under 18 years of age referred to the Trauma & Orthopaedics (T&O) department from 13/10/2023 until 01/11/2024 with a trampoline-related orthopaedic injury. Data extracted included age, sex, injury type, anatomical location, and management.

Results: From 3,025 paediatric referrals to T&O, 96 (3.2%) were trampoline-related. The mean age was 8.7 years old. The majority of patients were female (59.4%, n=57). Fractures constituted the majority of injuries (n=57). The ankle was the most common site of injury overall (27.1%, n=26), and for both fractures (n=14) and soft tissue injuries (n=12). The wrist and elbow were the next most common fracture sites, with similar frequency (n=11). The admission rate was low (2.1%, n=2); only one patient required surgical intervention. A clear bimodal seasonal variation was observed, with incidence peaking during April (n=14) and August (n=12).

Conclusions: This study provides contemporary UK data demonstrating that orthopaedic trampoline-related injuries, while common, predominantly result in minor fractures manageable conservatively. The ankle is the most common site. The strong correlation with seasonal spikes highlights a critical period for targeted public health initiatives and awareness campaigns to minimise risk. Despite the general minor nature of these injuries, thorough assessment is essential to recognise potential neurovascular compromise.

## Introduction

Trampolining has become a popular activity for children worldwide in recent decades. In the United Kingdom (UK) alone, trampoline sales have risen by 600% from 1997 to 2002 and have continued to rise by 50% annually thereafter [1]. This sharp increase in trampoline sales has continued over the last 15 years in the UK, with several indoor trampoline parks opening more recently in light of rising popularity and demand [2]. Furthermore, a rise in the number of home trampoline purchases was observed during the COVID-19 lockdown period, leading to a marked increase in home trampoline-associated injuries [3].

Various types and patterns of trampoline-related injuries have been reported, including soft tissue injuries, bony fractures, cervical spine injuries, focal neurology, and, in rare instances, fatalities [4-6]. The majority of these injuries occur in children aged 5 to 15 years old [5,7]. While the Royal Society for the Prevention of Accidents (RoSPA) has issued trampoline safety guidelines, concerns about the effectiveness of these voluntary measures exist as injuries continue to mount. Several international studies have described the epidemiology of and risk factors for these injuries, although much of this literature originates from North America, Australia, and Europe; limited relevant evidence has emerged from the UK recently, despite the rising incidence of trampoline-related pathology alongside its clinical and financial burdens [1].

This study investigated the incidences of and trends in varieties of trampoline-related orthopaedic injuries in paediatric patients referred to the Trauma & Orthopaedics (T&O) department serving two hospital sites across South West London, UK. The primary aim was to provide contemporary epidemiological data and injury profiles of paediatric patients presenting to UK Accident & Emergency (A&E) departments with trampoline-related trauma.

This article was previously presented as a poster at the Annual Sam Simmonds Meeting on May 9, 2025.

## Materials and methods

This retrospective observational study was conducted at Epsom and St Helier University Hospitals NHS Trust, comprising two district general hospitals, each with its own paediatric A&E departments that serve a population of approximately 497,000 patients across South West London and Surrey [8]. The aim of the study was to identify trends in trampoline-associated injuries in paediatric patients by anatomical site and season, determine the incidences of fractures versus soft tissue injuries by site, and illustrate the number of patients that required hospital admission for observation or surgical intervention versus conservative management as an outpatient.

The study population consisted of paediatric patients who sustained a trampoline-related injury. Inclusion criteria specified that patients must have been less than 18 years old and have been referred to the T&O department for a trampoline-related injury. Data collection took place for patients referred between 13/10/2023 and 01/11/2024 using Trust-wide electronic medical records. This involved compilation of patient age, sex, type of injury, anatomical location of injury, admission decision, and whether treatment was non-operative versus operative. Statistical analysis and figure generation were performed using Microsoft Excel (Microsoft® Corp., Redmond, WA, USA). Cases were identified by reviewing all paediatric Virtual Fracture Clinic (VFC) referrals and respective clinic outcome letters within the study period. These documents were compiled and manually screened for the mechanism of injury. Any patient for whom the word 'trampoline' was referred to in their clinical documentation was included in the study. This verbatim search was conducted using 'Clinical Manager iCM', the electronic medical records system utilised at the Trust where the study was conducted. An initial sample of the paediatric VFC referrals was screened independently by two distinct reviewers, who both identified the same cases appropriate for study inclusion, demonstrating the robustness and accuracy of the approach. Thereafter, the same methodology was applied by multiple independent reviewers to the rest of the sample population within the study period.

Formal ethics committee approval was not required based on this study's retrospective nature, combined with the fact that anonymised patient data was collected from existing Trust-wide electronic medical records with no compromise of patient confidential information or identifiable details.

## Results

**Figure 1:**
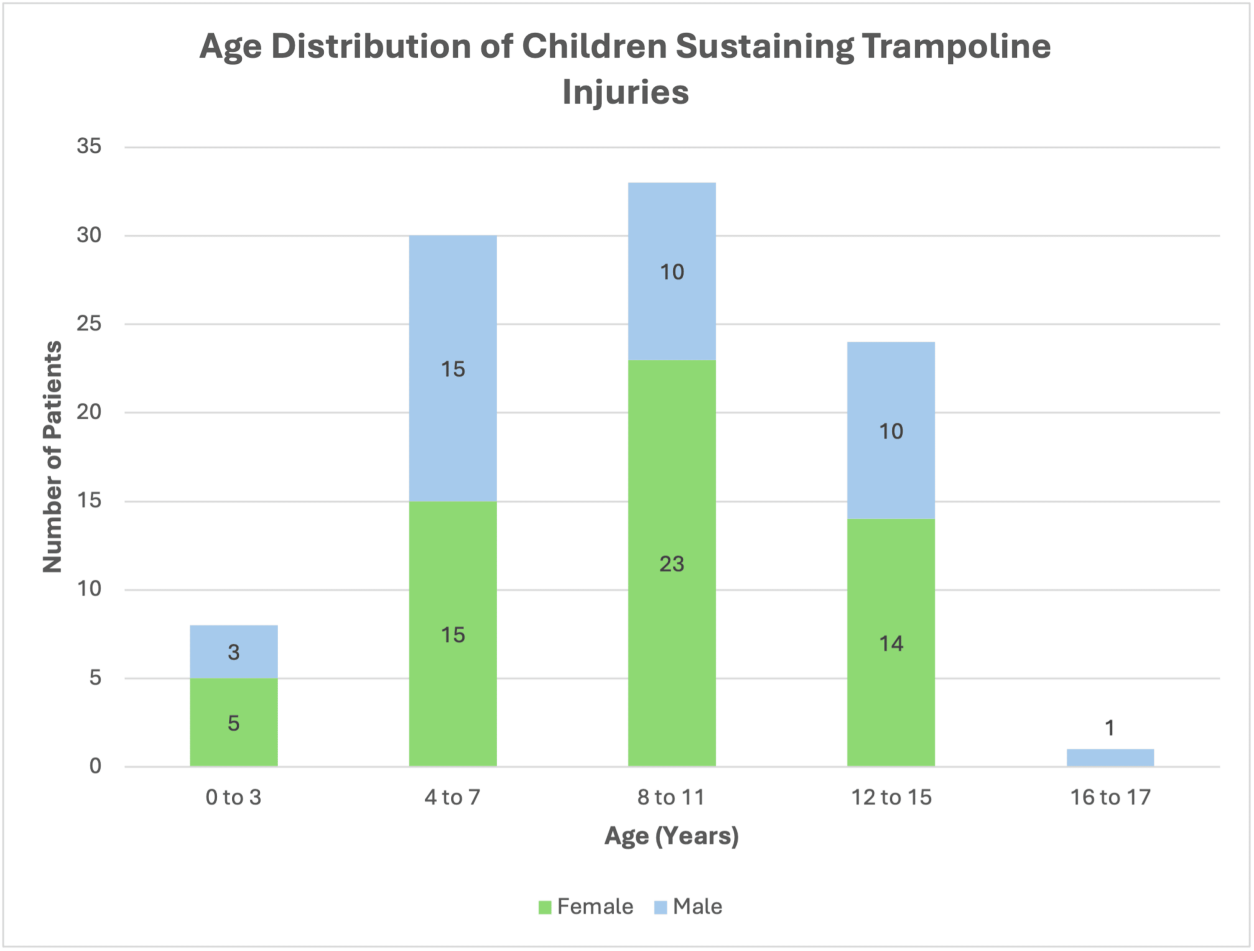
Distribution of ages by group for children sustaining trampoline injuries.

A total of 3,025 referrals for paediatric injuries were made to the T&O department at Epsom and St Helier University Hospitals NHS Trust over the aforementioned period. Ninety-six of these were for trampoline-related injuries and thus included in the study. The majority of patients were female (59.4%, n=57). Age at time of injury ranged from 1 to 17 years old, with an average age of 8.7 years old (Figure 1).

**Figure 2:**
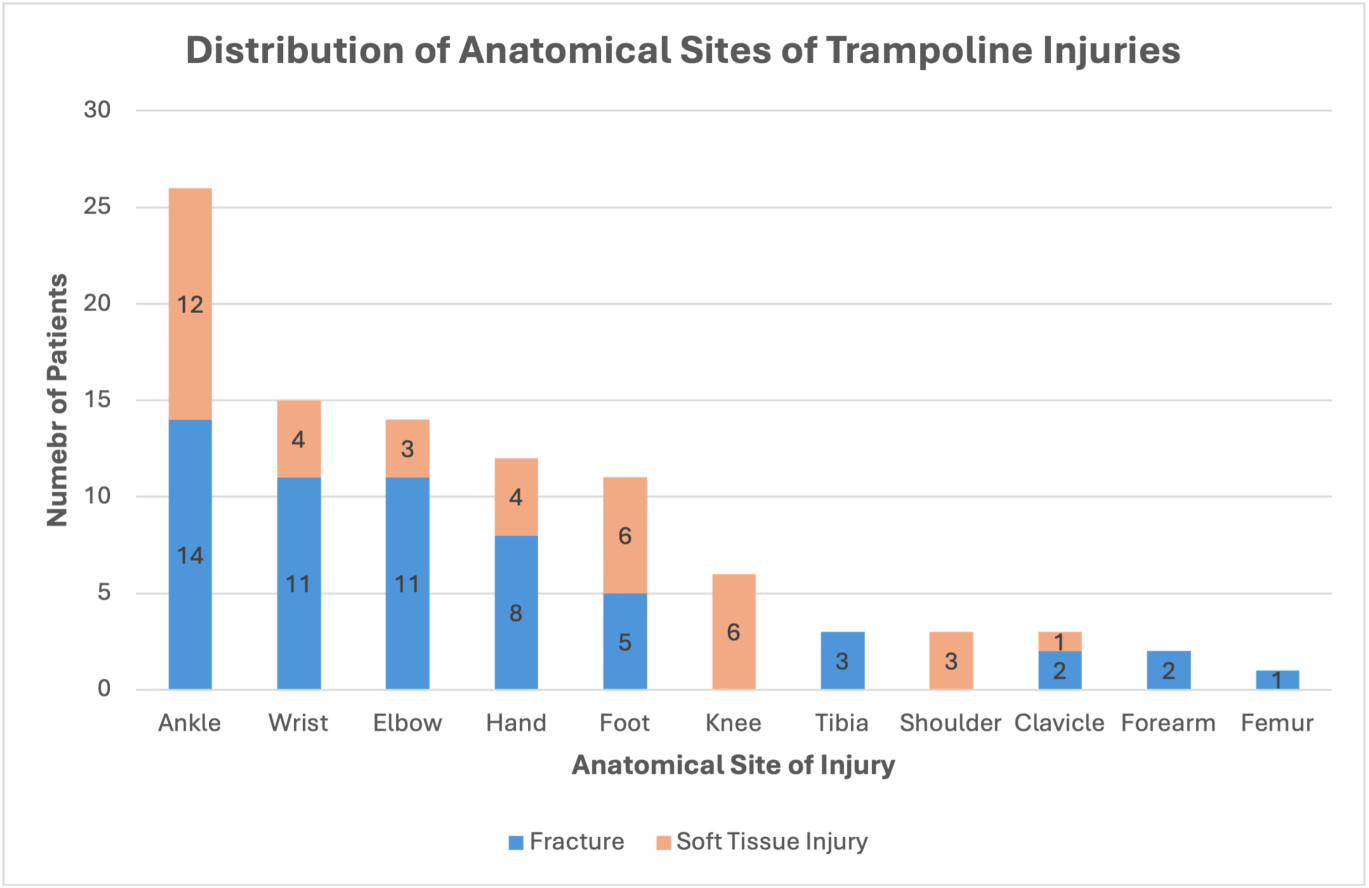
Distribution of trampoline-related injuries in children stratified by anatomical location and nature of injury (bony vs. soft tissue).

Ankle injuries accounted for 27.1% (n=26) of trampoline-related injuries observed in this study. A further analysis of these 26 cases revealed 14 fractures and 12 soft tissue injuries. The spectrum of fractures was diverse, including one combined distal tibia and fibula fracture, three distal fibula Salter-Harris type II fractures, one distal fibula Salter-Harris type I, one distal fibula physeal injury (unspecified Salter-Harris type), two medial malleolus avulsion fractures, one medial malleolus fracture, two distal tibia fractures, one anterior syndesmosis avulsion fracture, one distal tibia Salter-Harris type IV, and one Weber A fracture. All ankle injuries, including all fractures, were managed non-operatively with supportive care, such as immobilisation in a walking boot or cast, and follow-up as needed. Wrist injuries were the next most common presentation at 15.6% (n=15), followed by elbow injuries at 14.6% (n=14). Hand and foot injuries accounted for 12.5% (n=12) and 11.5% (n=11), respectively. Knee injuries occurred in 6.3% (n=6) of patients, whilst tibia, shoulder, and clavicle injuries each equally represented 3.1% of patients (n=3). Forearm injuries comprised 2.1% of patients (n=2). Finally, only one femur injury was observed (Figure 2).

Fractures were the most common injury pattern sustained (n=57). The ankle was the most commonly involved site of bony injuries (n=14), jointly followed by wrist (n=11) or elbow (n=11) (Figure 2). Regarding soft tissue injuries, ankles again remained the most common anatomical site of damage (n=12), followed by foot (n=6) and knee (n=6) injuries jointly as the next most common locations (Figure 2).

Two patients (2.1%) were admitted to the ward for a trampoline-related injury in this study. One admission was for prophylactic neurovascular observation of a distal humerus supracondylar fracture in a 10-year-old girl, due to the high-risk nature of this injury. The other was indicated for an eight-year-old boy with a radial head fracture requiring manipulation under anaesthesia and Kirschner wire fixation in theatre. Apart from that case, all other injuries were managed non-operatively.

**Figure 3:**
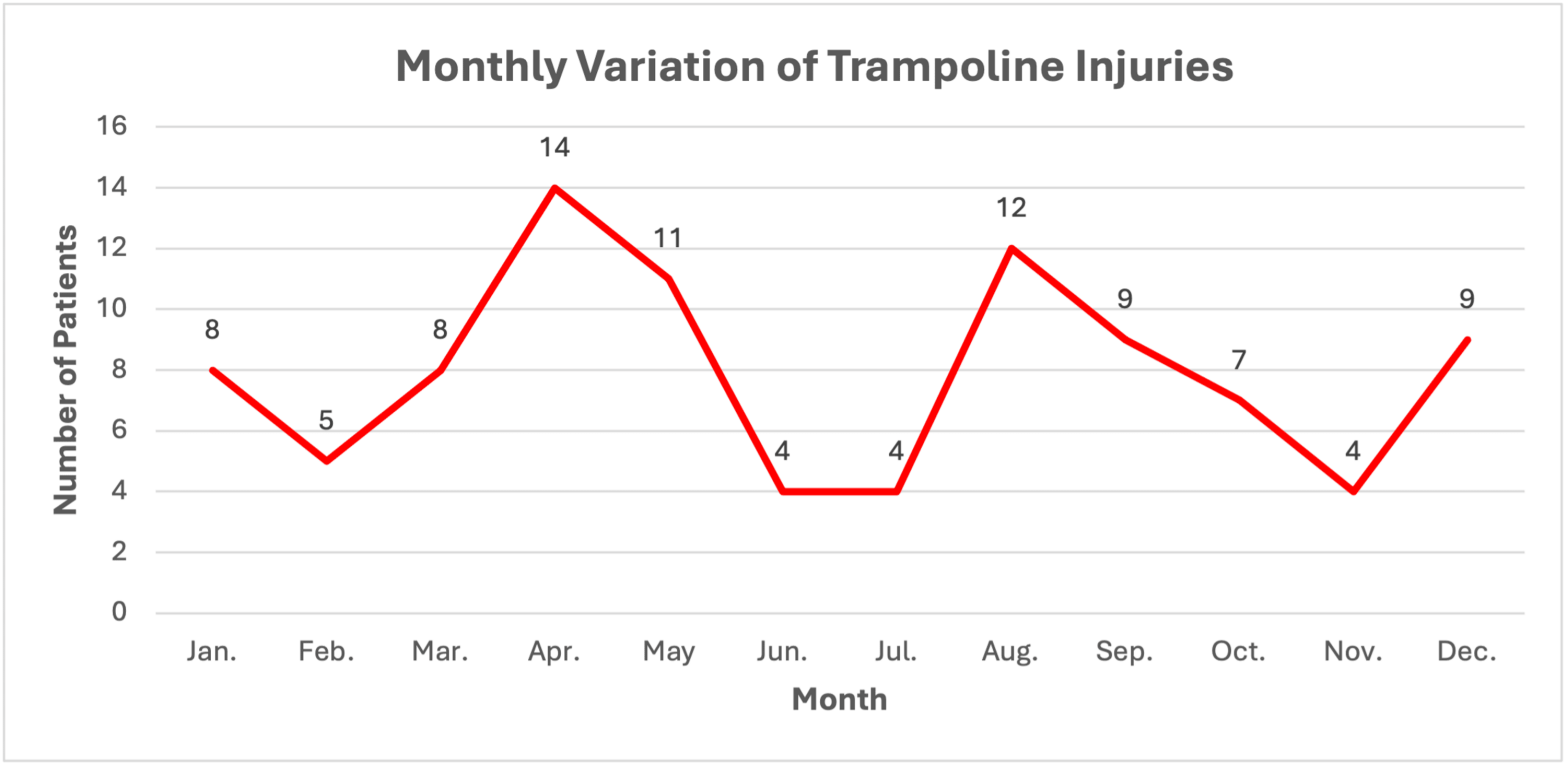
Distribution of trampoline-related injuries in children stratified by calendar month, illustrating data from 95 patients since one patient had a missing date of injury.

Trampoline injuries followed a bimodal distribution during the calendar year, with April observing the highest incidence (n=14), followed by August (n=12). June, July, and November exhibited the lowest incidences (Figure 3).

## Discussion

This study offers a unique, contemporary perspective on the epidemiology of paediatric trampoline-related orthopaedic injuries within a busy NHS Trust in South West London, England. Fractures were the most common type of injury sustained, with the ankle being the most commonly involved site, followed jointly by either wrist or elbow fractures. Similarly, the ankle again was the commonest anatomical site of soft tissue damage, followed by foot or knee injuries. Only two patients (2.1%) required admission, with one case simply being for monitoring of neurovascular status, whilst the other was the sole instance where operative management was indicated. A bimodal distribution in the calendar year was illustrated, with April observing the highest incidence of trampoline injuries, followed by August. While broader summer peaks have been previously reported, our study provides quantitative UK data that defines a more precise bimodal peak. This refined analysis is a key finding for targeting public health interventions.

These findings remain consistent with those of previous studies, highlighting that fractures were the most common pattern of injury, particularly of the ankle, wrist, and elbow [9], though notable differences exist. For instance, a North American study by Black and Amadeo (2003) reported a higher proportion of fractures (75%) [5]. Our UK data found a lower, though still predominant, fracture rate (59.4%). Furthermore, while the earlier study reported the upper extremity as the most common site (80% of orthopaedic injuries), our data highlights the ankle (27.1% of injuries) as the predominant site in our cohort. This difference in anatomical distribution of injury, from the upper extremity in previous international studies, to the lower extremity in our cohort, highlights a distinct pattern of trampoline-related orthopaedic trauma in our population and emphasises the clinical significance of ankle injuries in this high-energy mechanism.

The gender distribution (59.4% female versus 40.6% male) contrasts with previous literature, which typically reports that males were more frequently injured than females [10]. This variation may reflect local behavioural, demographic, and social factors, including participation rates and risk-taking behaviours across genders. The average age of injury identified in this study (eight years, seven months) remains consistent with previously published data, indicating that school-aged children remain the highest risk population [10].

A noteworthy finding resides in the seasonal variation in the incidence of trampoline injuries observed, peaking in April, followed by August. These months coincide with warmer weather and school holidays, likely leading to increased use of trampolines both at home and at recreational facilities [11]. This emphasises the importance of timing for public health campaigns, which should be launched in late March and late July to precede the April and August peaks. These campaigns could focus on safety guidelines (e.g., single-user rules, adult supervision) and could be disseminated through schools, GP practices, and in partnership with trampoline parks. It has previously been raised in the UK that current prophylactic measures are inadequate for raising parental and carer awareness of potential complications associated with trampolining [6].

Trampoline injuries in this study yielded a low admission rate (2.1%) and a high rate of non-operative management, consolidating the view that while common, the injuries are usually minor and manageable in an outpatient setting. However, the two cases requiring admission highlight the potential severity of these injuries in terms of neurovascular compromise and functional deficit if not corrected in a timely manner, necessitating vigilance during initial clinical assessment. These rates are significantly lower than those from international studies; in Australia, admission and operation rates for paediatric trampoline injuries have been reported as high as 21% and 18%, respectively [12]. The notably lower admission and operation rates in our study are likely due to the specific nature of our cohort, which captured injuries referred to an orthopaedic service, thereby excluding more severe non-orthopaedic injuries (e.g., major head or abdominal trauma) that would be admitted under other specialities and contribute to higher overall admission rates in more inclusive studies.

The limitations of this study firstly include that it was conducted at a single NHS Trust, although this was ameliorated by virtue of the Trust having two separate paediatric departments at distinct sites serving a large local population. Furthermore, the retrospective nature of the study limits the ability to inform on the causality of findings and relies on the accuracy and completeness of clinical documentation. In addition, the study may underrepresent certain complex injuries, such as those to the paediatric elbow, which can be difficult to diagnose on initial radiographs by non-specialists due to the predominantly cartilaginous anatomy. This may have contributed to fewer referrals to the T&O department and potentially missed musculoskeletal (MSK) injuries. Additionally, the data from this study did not allow for a distinction between injuries sustained on home trampolines (typically outdoors) versus those at commercial indoor trampoline parks due to a lack of specification on VFC referrals. This is an important factor, as the injury mechanisms and severity of injuries may differ between these environments. Future studies should document the injury environment prospectively to explore this variable. Future multi-centre studies across the UK are necessary to corroborate these trends on a national scale and identify if such limitations are universal in order to identify potential solutions and prevent MSK injuries from being missed.

## Conclusions

Trampolining and its associated injuries in children have risen significantly in the UK and worldwide in recent years, generating considerable clinical and financial burdens for the NHS in the UK. This study provides a contemporary insight into the rising incidences of and trends in paediatric trampoline-associated orthopaedic trauma in the UK. Ankle injuries were the most common site of bony or soft tissue trauma observed. Although the majority of injuries were managed non-operatively, careful scrutiny of neurovascular status and anatomical function remains essential to reduce the risk of limb-threatening neurovascular compromise or permanent functional deficit if not appropriately managed in a timely manner. Seasonal spikes in trampoline-related pathology in the spring and summer necessitate well-timed public health campaigns and resource planning for emergency care providers. Future work involving multiple centres and collaboration between A&E and T&O departments is indicated to further improve understanding of trampoline injury patterns, assess the impact of preventive measures, and ultimately guide policymaking to combat the rising clinical and financial tolls associated with trampoline-related injuries.
